# Bibliometric Analysis of Papaya and Dragon Fruit By-Products

**DOI:** 10.3390/foods14132275

**Published:** 2025-06-26

**Authors:** Noreima Barroso-Torres, M. Gloria Lobo, Eva Dorta

**Affiliations:** Department of Crop Production in Tropical and Subtropical Areas, Instituto Canario de Investigaciones Agrarias, 38270 Tenerife, Spain; nbarroso@icia.es (N.B.-T.); globo@icia.es (M.G.L.)

**Keywords:** *Carica papaya*, *Hylocereus* spp., bioactive compounds, waste

## Abstract

Tropical fruits have become increasingly popular due to their interesting nutritional composition. This rise in their consumption has resulted in more by-products generated during their production, processing, and commercialization. Papaya, for instance, is one of the leading tropical fruits produced globally due to its essential macro- and micronutrients for a healthy diet. On the other hand, dragon fruit, less known worldwide, is gaining popularity due to its nutrient and bioactive compound content. This review uses bibliometric analysis as a tool to investigate the scientific literature related to these two fruits, focusing specifically on their by-products. The objective is to identify the key authors and countries that are making substantial contributions to the research on these fruits and their by-products, such as peels and seeds. It will provide an overview of existing topics and highlight areas needing further investigation to enhance understanding and practical applications. This approach will help guide future research and innovations related to these fruits and their potential uses.

## 1. Introduction

The agro-industrial sector currently generates significant amounts of by-products and waste. According to the Food and Agriculture Organization (FAO), approximately one-third of global food production, equivalent to 1.3 billion tons, is discarded. In recent years, the consumption of processed fruit products such as juices, nectars, and frozen fruits has increased, resulting in a rise in the generation of by-products, principally peels and seeds [1].

Tropical fruits, while representing only 3% of global agricultural trade, have experienced a substantial increase in production driven by rising international demand [2]. Market analyses conducted by the FAO indicate that the production and consumption of tropical fruits have risen by 9% over the past decade [3].

The consumption of fruits exerts a positive effect on human health due to their rich nutritional composition. Fruits are an excellent source of dietary fiber, essential vitamins, such as vitamin C and provitamin A, minerals, like potassium and magnesium, and a wide variety of bioactive compounds, including polyphenols, flavonoids, and carotenoids [4,5]. These compounds have been associated with various health benefits, such as antioxidant, anti-inflammatory, and cardioprotective properties, as well as a reduced risk of chronic diseases, like cardiovascular conditions and certain types of cancer [6].

Consequently, the increasing demand for fruit-based products, including juices, purees, and dehydrated fruits, has led to the large-scale processing of these commodities, thereby generating substantial amounts of residues such as peels, seeds, and pulp fibers. These by-products, often discarded as waste, retain a significant proportion of the fruit’s bioactive compounds, making them valuable raw materials for further applications. Given their potential, the valorization of fruit by-products presents an opportunity to develop functional food ingredients and nutraceuticals while simultaneously addressing environmental concerns associated with food waste [7].

There is a global trend that emphasizes not only food security but also the nutritional quality of the food we consume. In this context, fruits play a crucial role in the diet, as they provide essential vitamins, minerals, and bioactive compounds necessary for the proper functioning of the body while meeting quality and safety standards [8]. Bioactive compounds have garnered increasing attention from both the scientific community and consumers due to their remarkable health benefits. These secondary metabolites, typically present in low concentrations, serve as precursors in various physiological functions. Numerous studies have demonstrated their impact on human health [9].

Fruit by-products are abundant in bioactive compounds, such as flavonoids, phenolic compounds, carotenoids, and anthocyanins, which are associated with several health benefits, including the inhibition of oxidative stress, the regulation of metabolism, the prevention of microbial infections, and the reduction in the risk of certain diseases, among others [10].

Major tropical fruits such as mangoes, pineapples, avocados, and papayas dominate the market, alongside bananas, which lead in global production volume. These fruits are not only internationally recognized but also widely marketed and consumed across the globe [11]. In contrast, the production of lesser-known tropical fruits, regarded as novelties in the global market, is significantly smaller. The aforementioned exotic fruits, including guava, lychee, passion fruit, dragon fruit, mangosteen, longan, rambutan, and jackfruit, warrant increased attention due to their distinctive flavors and significant market potential. These fruits present unique opportunities for exploration and development within the agricultural and culinary sectors. These fruits are important sources of fiber, polysaccharides, vitamins, organic acids, and minerals. Beyond the conventional nutrients, they encompass diverse bioactive compounds, such as phenolic compounds and other phytochemicals that contribute significantly to overall health improvement and disease prevention.

Despite their nutritional richness, several compounds present in these fruits remain underexplored. For example, betacyanins and betaxanthins—betalain pigments found predominantly in the peel of pitahaya—have attracted interest for their antioxidant and anti-inflammatory properties, yet the current literature remains limited. Likewise, recent studies indicate the presence of enzymatic antioxidants and lipid-soluble pigments in papaya peel and seeds that have not been fully characterized. Therefore, a deeper understanding of the composition of their by-products is needed to better harness their potential for functional food and nutraceutical development.

Papaya (*Carica papaya* L.), which belongs to the Caricaceae family and is native to southern Mexico and Central America, ranks third in the tropical fruit market, following mango and pineapple. Its production is reported to be 185.95 tons every three years [12]. This tropical fruit is rich in carotenoids, phenolic compounds, fiber, minerals, and vitamins, which contribute to its classification as a functional food [13,14]. The nutritional properties of papaya have been studied for decades. Papayas can be consumed fresh in fruit salads, blended into juice, or processed into jams, jellies, candies, chips, etc. [15]. The increasing demand and recognition of papaya, owing to its distinctive flavor and characteristics, have led the international market to establish specific criteria regarding size, shape, weight, and standards of nutritional and organoleptic quality [16]. Consequently, fruits that do not meet these specified standards are often rejected, resulting in substantial waste. Additionally, the harvesting process generates a considerable amount of by-products due to field damage, the occurrence of fruits at various stages of ripeness, and losses incurred during harvest. The by-products, specifically the peel and seeds, comprise roughly 8.5% to 12% of the total fruit mass and are nutrient-dense, providing a variety of macro- and micronutrients critical to human nutrition. The peel is a noteworthy source of carotenoids, dietary fiber, and phenolic compounds, which contribute to its antioxidant properties and potential applications in the food and pharmaceutical industries [17]. Seeds have been identified as a considerable source of lipids, carbohydrates, and essential fatty acids, including oleic acid, palmitic acid, and linoleic acid, among others [18].

The substantial waste generated during the processing of papayas presents an opportunity for recovery and repurposing into value-added products [19].

Dragon fruit (*Hylocereus* spp.), also known as pitahaya or pitaya, is the fruit of a climbing plant of the genus Hylocereus, which belongs to the Cactaceae family [20]. The global market for dragon fruit is experiencing robust growth. In 2024, global dragon fruit production reached approximately 2 million tons, with Vietnam leading as the top producer, followed by China, Indonesia, Mexico, and several other Latin American countries. In Spain, production remains limited, with an estimated cultivated area of around 20 hectares, though initiatives are underway to expand its commercial viability.

Pitahaya fruits are classified as functional foods due to their high nutritional content and nutraceutical properties, including vitamins (C, B1, B2, B3), carbohydrates, proteins, minerals, and fiber, as well as bioactive compounds such as flavonoids, anthocyanins, carotenoids, and polyphenols [21]. It has been observed that the skin of pitahaya contains a higher concentration of bioactive compounds compared to the pulp. Additionally, the seeds are significant in terms of oil composition, as the oil extracted from pitahaya seeds contains high amounts of essential fatty acids. The skin is one of the most important by-products in the processing of pitahaya to produce juices and ready-to-eat products, being rich in essential nutrients, including proteins and carbohydrates. The skin of pitahaya is used as a functional ingredient in foods due to its betalain content [22].

Bibliometrics is a valuable method for evaluating scientific output by analyzing published documents. This approach assesses key authors, institutions, keywords, and citation patterns within a specific research domain. Through bibliometric analysis, researchers can uncover gaps in the existing literature, identify emerging topics, and explore future directions for scientific inquiry. To foster sustainable development and implement waste valorization techniques in the agro-food industry, it is crucial to identify research areas that require further exploration [23]. Consequently, it is essential to review and update current studies, focusing on the researchers involved, the subjects being examined, and all aspects related to the extraction of bioactive compounds from tropical and subtropical fruit by-products, particularly papaya and pitahaya, given their increasing relevance.

In this context, this study aims to address the following research questions: Which countries and institutions are leading scientific production in this area? What specific bioactive compounds—such as carotenoids, betalains, and polyphenols—are most frequently studied in papaya and pitahaya by-products? What are the predominant extraction techniques used for their recovery? These questions allow for a comprehensive understanding of current research trends and gaps that still need to be addressed.

This study aims to provide a current review and bibliometric analysis of scientific research on pitahaya and papaya, focusing on their by-products.

## 2. Materials and Methods

**Figure 1:**
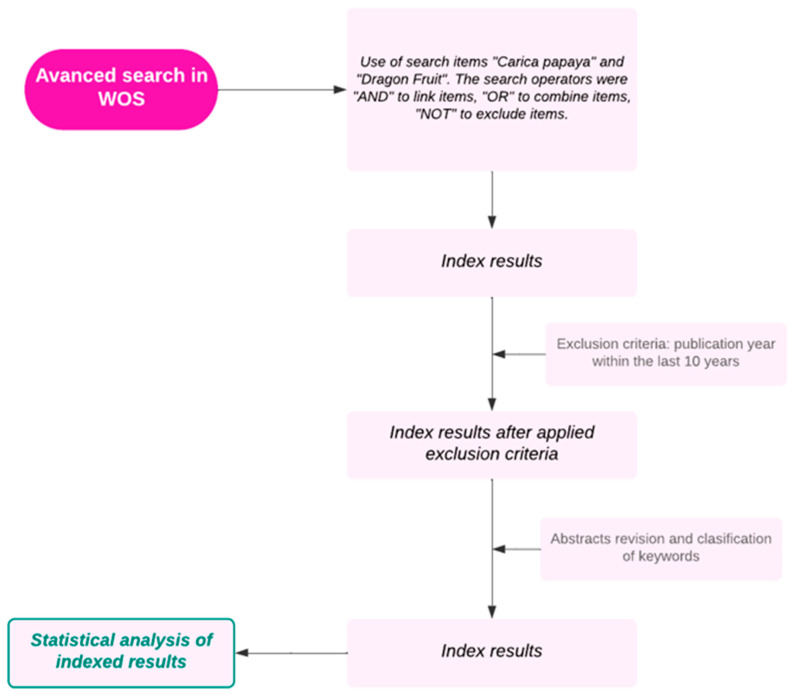
Flow diagram of data collection.

The bibliometric analysis was conducted based on the methodology established and reported in previous studies [24]. The flow diagram detailing the steps undertaken for the bibliographic search and bibliometric analysis is presented in Figure 1. To reduce the possibility of errors and ease the integration and analysis of data with different software, we used one database only. Thus, Web of Science © (WOS) was chosen because it is a leading database of abstracts and citations of the literature that have been peer-reviewed. The documents included in this analysis consist of scientific articles, theses, patents, and books, all of which are written in English.

### 2.1. Data Collection

This bibliometric study incorporated scientific data from the Science Citation Index Expanded (SCI-E) of ISI Web of Science ©. This database was selected for its high indexing standards, widespread academic use, and ability to provide consistent, high-quality records suitable for bibliometric analyses, thereby minimizing duplication and inconsistencies in sources. We based the document retrieval on the Prisma protocol [25] and retrieved the documents from 2014 to the present using the search key “Papaya,” “*Carica papaya*,” or “*Carica papaya* L.” for papaya and “*Hylocereus polyrhizus*,” “pitahaya,” “dragon fruit,” or “pitaya” for dragon fruit. The research examined the occurrence of terms in the title, abstract, and keywords of the studies, and we did not establish a time frame of knowing when. The operators that the search used were “AND” to link items and “OR” to combine terms effectively. The search was limited to complete articles to maintain a standardized data set, facilitating operationalization and subsequent analysis. We eliminated three duplicate articles. The titles, abstracts, and data from the articles were read during the selection process.

### 2.2. Data Analysis

The advancement of bibliometric software has enabled the analysis of scientific fields, topics, and areas of interest through mathematical algorithms. These programs process bibliometric data using statistical methods, including word count laws, co-citation analysis, bibliographic coupling, and keyword co-occurrence, among other techniques [26]. VOSviewer software (version 18) was selected due to its suitability for keyword analysis and strategic map generation.

VOSviewer software enables the creation of networks that connect concepts, terms, and keywords. The visualization of the similarity (VOS) algorithm generates VOSviewer maps, where the distance between objects accurately represents their similarity based on mathematical precision [27]. This feature was utilized in this study to construct co-occurrence networks using keywords. The keywords assigned by authors form nodes, with node size indicating the frequency of keyword occurrences. Additionally, the links between nodes are proportional to the co-occurrence of the terms [28].

## 3. Results

Papaya has been a topic of study since the mid-1980s, when the annual number of publications began to exceed 50. The most significant increase in publications occurred between 2000 and 2010, during which the average number of studies related to papaya rose by approximately 28.0%. Since then, the number of publications on papaya has remained relatively constant. This stabilization may be attributed to the thematic maturity of the field. While initial growth was driven by increasing interest in functional foods and the bioactive compounds present in papaya, subsequent research has focused on more specific and applied aspects, including by-product valorization, advanced extraction techniques, and nutraceutical applications. Interest in dragon fruit (*Hylocereus* spp.) was first documented in 1982. However, it was not until 2005 that research activity significantly increased, with fewer than 20 studies published globally by that year. This number doubled by 2014, indicating a growing scientific focus on this tropical fruit.

**Figure 2:**
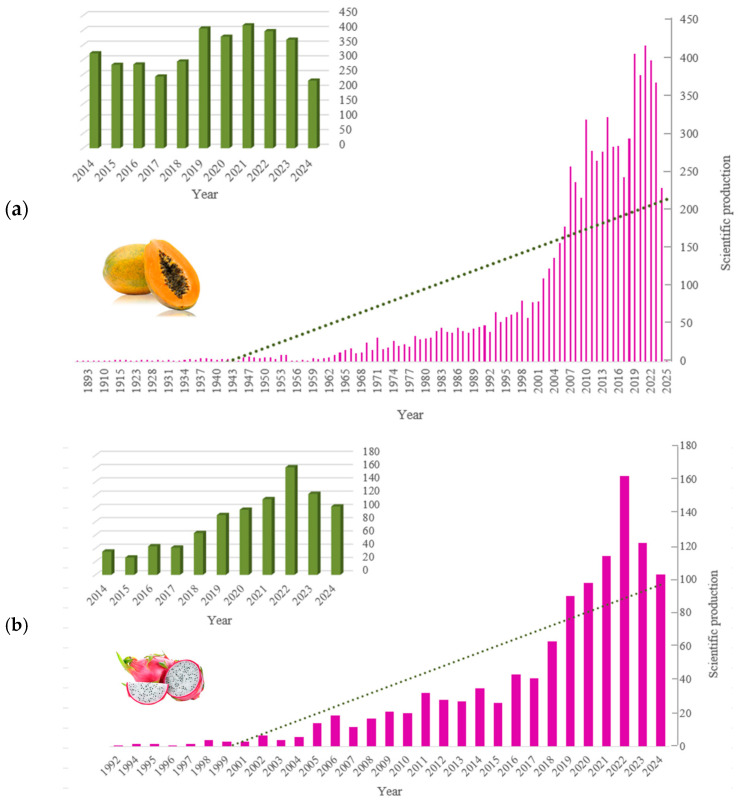
Trends in the number of scientific publications over time for (**a**) papaya and (**b**) dragon fruit.

Over the past decade (2014–2024), a total of 19,283 publications were indexed for papaya, compared to 1267 for dragon fruit. This indicates that there have been 93.4% more publications about papaya than about dragon fruit. The average number of annual publications for papaya remained relatively consistent (362 publications per year), demonstrating that it has been a fruit of significant interest to the scientific community. Dragon fruit publications have exhibited a steady upward trend over the observed period, notwithstanding some fluctuations (Figure 2). Thus, between 2018 and 2024, the number of publications increased by 28.6%. Research on papaya peaked between 2019 and 2022, with the highest scientific output occurring in 2021 and 2022, accounting for 10.5% of the total.

To quickly understand the main contents and conclusions of research, it is relevant to know the characteristics and origin of the journal in which it has been published [24].

The VosViewer program identified the areas with the highest publication rates, which included Plant Sciences, Agriculture, and Food Science Technology for both fruits. The fourth and fifth most significant research areas were Biochemistry Molecular Biology and Environmental Sciences Ecology for papaya, and Physiology and Genetics Heredity for dragon fruit (Table 1).

The top five journals in the productivity metrics for papaya and dragon fruit for the last decade are shown in Table 2, as they have the highest number of publications in the field of these fruits. These journals consistently address research themes that fall under the specialized categories of food science and agricultural studies.

Keywords play a crucial role in defining the research topics within a specific field and encapsulating the main content of scholarly articles. They enhance the search experience for authors, making it easier to locate relevant studies. Additionally, analyzing keywords can provide valuable insights into emerging research trends, allowing for informed predictions and direction of studies in each area. This makes keywords important not only for individual searches but also for understanding the evolving landscape of knowledge within a discipline [24].

**Figure 3:**
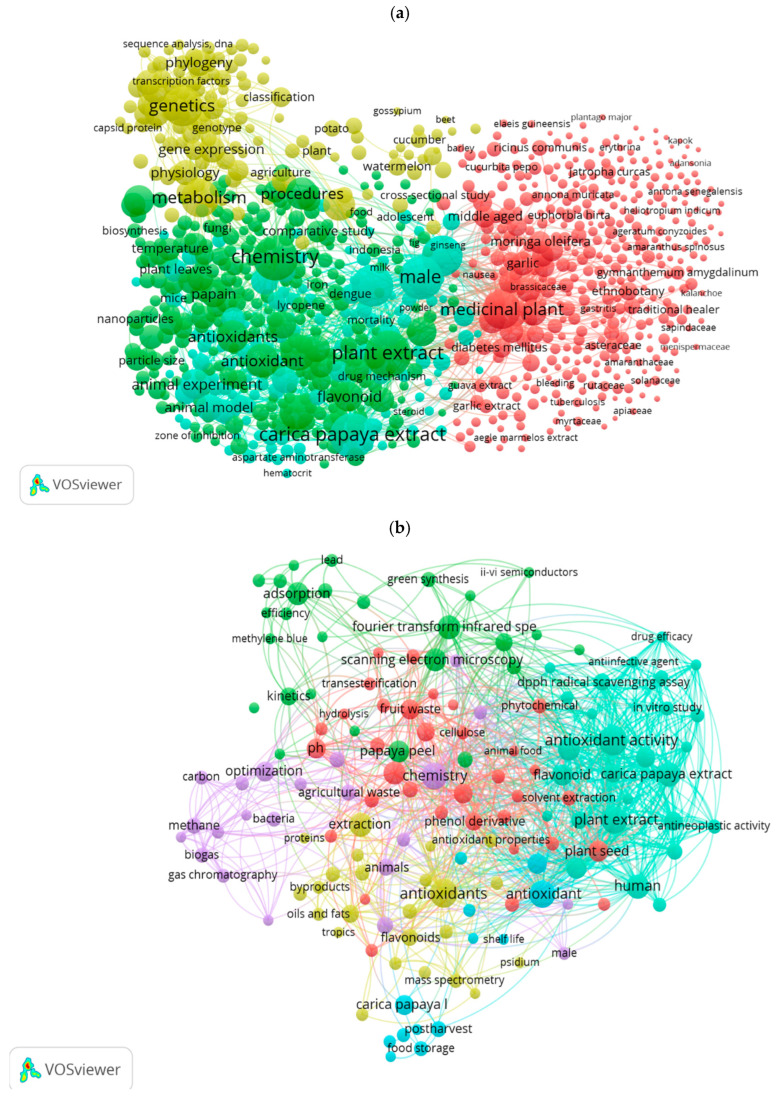

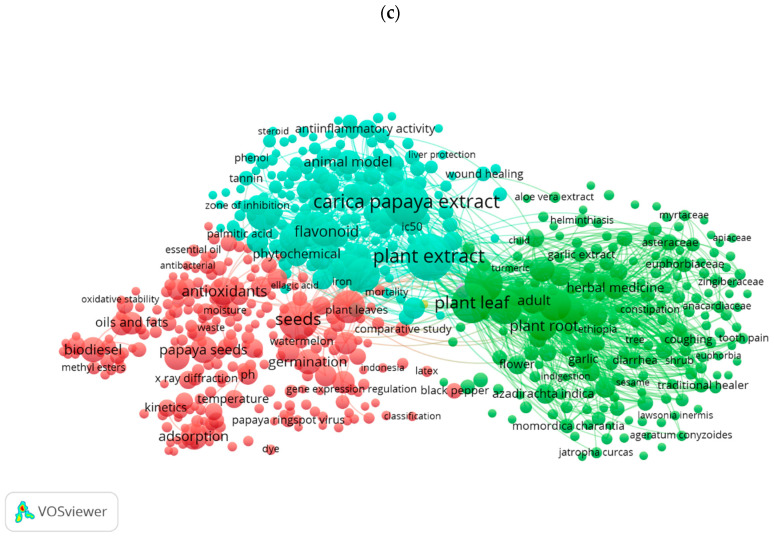
Diagrams showing the co-occurrence of keywords for searches conducted using the chosen item or combinations of items: (**a**) “papaya”, (**b**) “papaya” AND “peel”, (**c**) “papaya” AND “seed”.

The co-occurrence analysis of keywords utilizes the Louvain clustering algorithm to effectively illustrate the frequency of keyword occurrences, with the size of each item on the map reflecting its level of prevalence. Additionally, distinct colors are employed to categorize the keywords into various macro-area groups, thereby enhancing the overall clarity and comprehensibility of the analysis. In the present study, for searches regarding papaya in general, four areas encompassing the identified keywords are observed, with the most significant terms related to “botany”. There are also two major groups associated with the biological functions attributed to papaya consumption and the extraction of compounds from it. A smaller macro-area, characterized by a lower number of keywords but closely linked to the two, classifies terms related to genetics and physiology (Figure 3a). In the specific context of searches on papaya by-products, it is observed that the categorization of terms related to the peel is less distinctly outlined. The leading category primarily includes keywords associated with “antioxidant activity” (Figure 3b). The diagram derived from the literature on papaya seeds reveals three distinct macro-areas. The most prominent area highlights keywords associated with “medicinal applications”. In contrast, the other two areas focus on the “chemical composition”: one on oils and fatty acids, while the other emphasizes phytochemicals and bioactive compounds (Figure 3c). Terms that occur together in the same article are represented by connections between them, and the thickness of these connections indicates how frequently the linked terms occur together.

**Figure 4:**
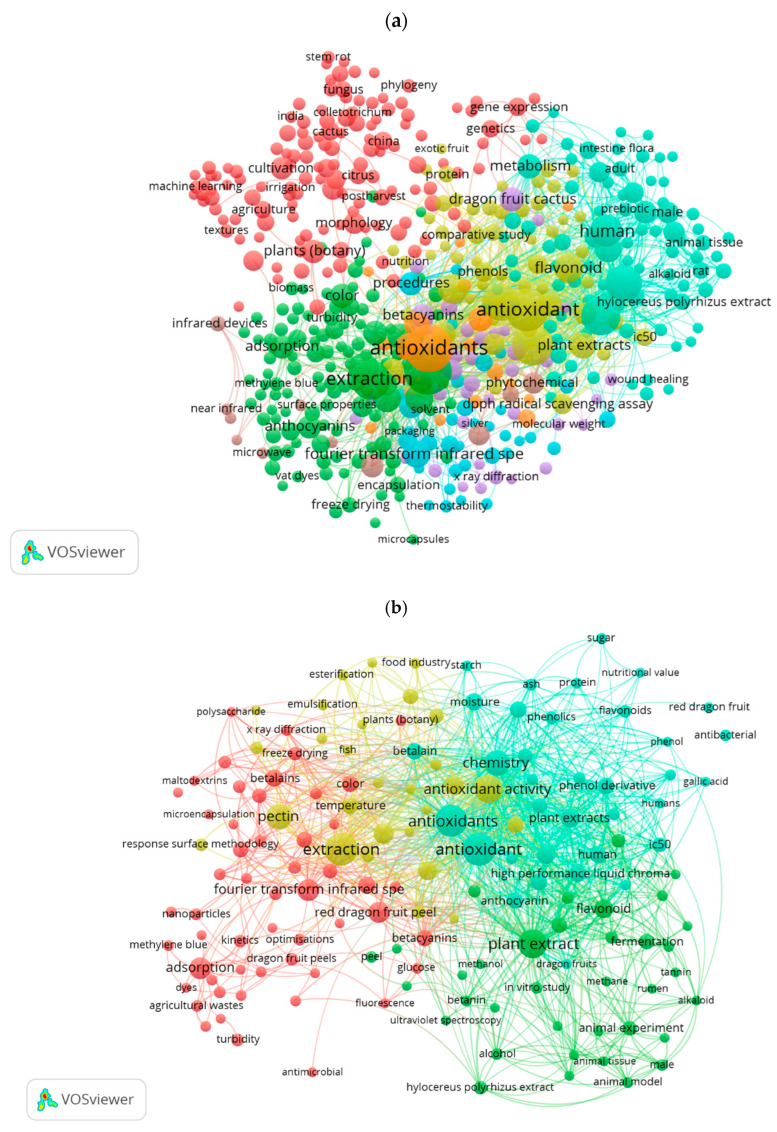

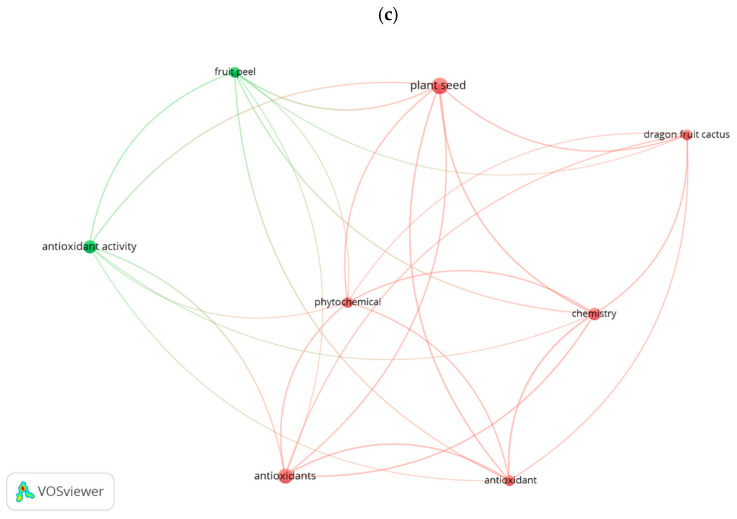
Diagrams showing the co-occurrence of keywords for searches conducted using the chosen item or combinations of items: (**a**) “dragon fruit”, (**b**) “dragon fruit” AND “peel”, (**c**) “dragon fruit” AND “seed”.

The keywords co-occurrence analysis found in the bibliographic analysis focusing on dragon fruit and its by-products reveals a network of numerous macro-areas for “dragon fruit” in general. Among these, articles that include terminology related to “biological functions” are particularly prominent, along with another area involved in the cultivation, field management, and harvesting, and a third that pertains to terms dealing with “organoleptic quality” and “chemical composition” (Figure 4a).

When examining dragon fruit by-products, specifically the peel, a clear pattern emerges regarding keywords associated with investigating “antioxidant activity” and the “relevant compounds” (Figure 4b). The final graphic representing dragon fruit seeds clearly shows a much lower quantity of terms when compared to the other searches, offering limited insights into keyword patterns in this area (Figure 4c). The scarcity of keywords related to dragon fruit seeds indicates a limited body of literature on this specific topic. The lack of extensive research underscores a potential gap in scientific knowledge, making it an intriguing and promising area for further investigation. Examining the bioactive compounds, nutritional properties, and potential applications of dragon fruit seeds could provide valuable insights and enhance the broader understanding of their functional and industrial potential.

When analyzing the conceptual maps generated from the co-occurrence of keywords, notable patterns emerge for both papaya and pitahaya. The first diagram for papaya reveals a relatively limited presence of keywords related to genetic studies, indicating that this area has not been extensively explored. Conversely, the maps associated with papaya by-products, such as the peel and seeds, showcase complex and well-connected keyword networks, reflecting a greater research focus on the compositional analysis of these by-products, particularly regarding their bioactive compounds. However, there appears to be a reduced volume of studies focusing on in vitro and in vivo evaluations of the biological activities linked to these compounds, pointing to an opportunity for future research. In terms of pitahaya, the diagram highlights a clear research trend centered on investigating its antioxidant activity. While this demonstrates a growing interest in one of its key bioactivities, it also reveals a gap in exploring other biological effects and the specific bioactive compounds responsible for them. Furthermore, the conceptual map corresponding to pitahaya seeds is comparatively simple and sparse, underscoring the lack of scientific literature available on this by-product. This presents a promising research avenue for future studies to unlock the potential of dragon fruit seeds.

**Figure 5:**
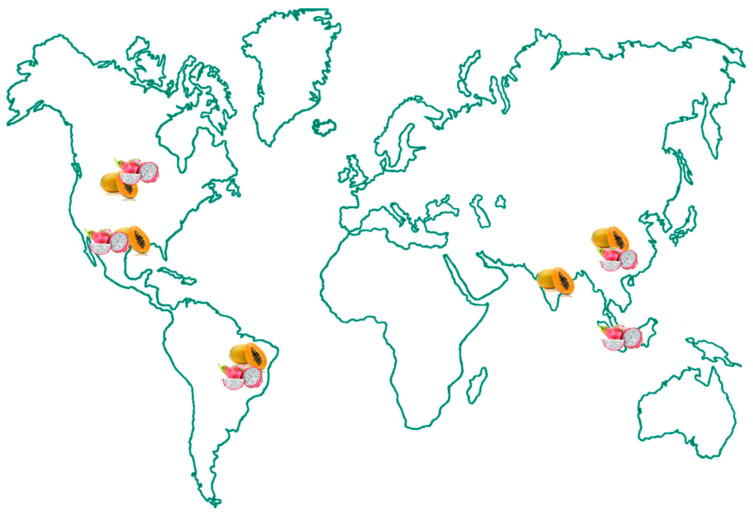
Distribution of the scientific production of dragon fruit and papaya in the world’s most significant nations.

The analysis of productivity based on the total number of publications by country (Table 3) reveals that the United States is the foremost contributor to the body of research on papaya, generating 11.46% of the overall publications. Then, it is followed by India and Brazil in terms of research output. The leading nations of scientific production for dragon fruit are China, Brazil, and Mexico, as illustrated in Figure 5. When examining dragon fruit, the United States positions itself fifth, contributing 11.60% of the total publications. Brazil and Mexico are very important contributors in scientific publications for both fruits.

This geographical disparity, particularly the dominant position of China in dragon fruit research compared to its lower output in papaya studies, can be largely attributed to regional cultivation patterns and national research priorities. In recent years, China has significantly expanded the cultivation and commercialization of dragon fruit. This expansion has been supported by strategic investments and government programs, promoting both agricultural development and scientific research on dragon fruit. In contrast, papaya has long been cultivated in tropical regions, such as India, Brazil, and Mexico, where it holds greater traditional and economic relevance. These countries have historically led papaya research due to their established production systems and institutional focus. Consequently, the observed differences in scientific output reflect broader trends in crop prioritization, funding allocation, and agricultural strategy at the national level.

**Figure 6:**
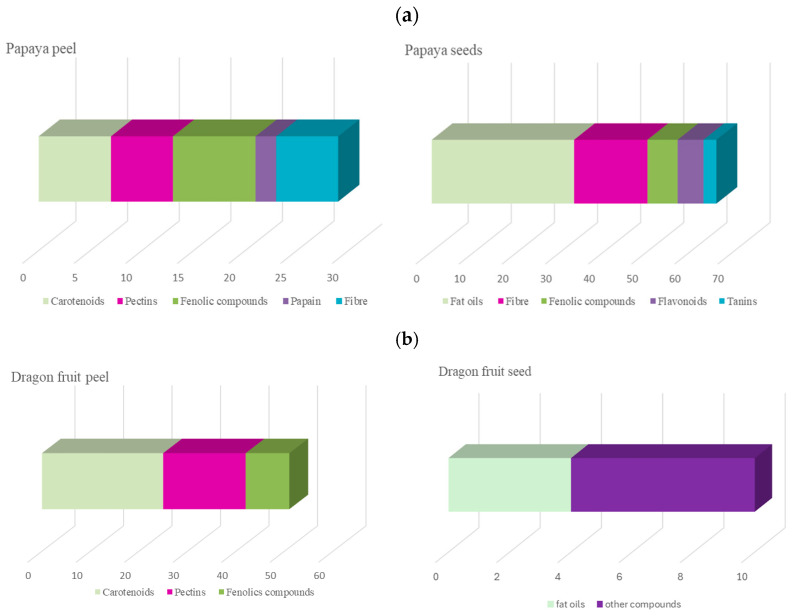
Main compounds studied in the by-products of (**a**) papaya and (**b**) dragon fruit.

Conversely, there was a limited search and bibliometric analysis of publications about fruits’ by-products. Only those publications that mentioned the by-products (skin, pulp, and seeds) out of all the previously examined publications were indexed for this purpose. It is observed that by-products of papaya are mentioned in 9.7% of all papaya publications, compared to 29.0% of dragon fruit publications. Regarding the main themes encompassed in the study of papaya by-products, it was observed that the scientific production focuses most on agriculture, field management of these fruits, postharvest activities, and genetic improvements. Only 17.5% and 10.9% of the publications on papaya by-products primarily address the study of their composition and biological activities for peel and seeds, respectively (Figure 6a,b).

The most studied bioactive compounds in the dragon fruit peel include phenolic compounds, carotenoids, and pectins, accounting for 14.0%, 12.0%, and 10.0% of the total publications in this field, respectively (Figure 6b). The seeds’ oil and fatty acid profiles have garnered the most scientific attention in the past decade, accounting for 37.0% of the publications on their composition. There has been significantly less research output regarding pitahaya by-products in terms of the number of publications (n = 188). However, the majority can be categorized within the field of studying their phytochemical composition (39.4%).

Despite the recognized nutritional and functional value of fruits, research on the bioactive compounds in their by-products remains limited, particularly for underutilized or emerging species. This gap highlights the need to explore the potential of fruit residues as valuable sources of health-promoting compounds. In this context, the transition toward a circular economy has gained significant momentum within the agri-food industry, driven by the need to reduce environmental impact and enhance resource efficiency. This model promotes the revalorization of by-products and waste generated throughout the food supply chain, fostering sustainability and innovation in food systems. In alignment with the United Nations Sustainable Development Goals (SDGs), particularly Goal 12.3, which aims to halve global food waste by 2030, the valorization of fruit by-products has become an area of increasing scientific and industrial interest. Additionally, the European Union has enacted legislative measures such as the Circular Economy Action Plan and the Waste Framework Directive to minimize food waste and encourage the sustainable use of biological resources. In this context, fruit by-products have drawn attention due to their rich content of health-promoting compounds. Peels, seeds, and pomace often retain a significant concentration of bioactive compounds, which are frequently more abundant in the by-products than in the edible portions of the fruit.

**Figure 7:**
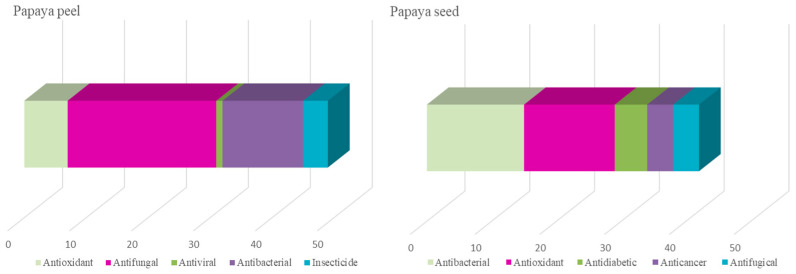
Biological activities mainly studied in papaya by-products.

For the pitahaya peel, the main compounds studied over the past decade have been carotenoids, anthocyanins, and pectin, accounting for 34.0% and 23.1% of the total publications in this field, respectively (Figure 7). Regarding the seeds of this fruit, it is noteworthy that limited literature is available, primarily focusing on genetic improvement, cultivation techniques, and field activities. In the study of their composition, over 50.0% of the publications aim to investigate the fatty acid profile of this by-product.

## 4. Conclusions

This review highlights the significant potential of papaya and dragon fruit by-products, particularly peels and seeds, as valuable sources of nutrients and bioactive compounds. The growing number of scientific publications reflects the increasing interest in these residues due to their possible applications in the agri-food, pharmaceutical, and cosmetic industries. However, the current literature reveals notable gaps, especially concerning in vitro and in vivo studies exploring the biological activities of these by-products. Most existing research focuses on antioxidant, anti-inflammatory, anticancer, and antidiabetic activities, yet few studies examine the direct effects of specific extracts, concentrations, or freeze-dried formulations derived from peels and seeds.

Within the scope of bioactive compound analysis, most studies are limited to quantifying general groups—such as phenolics, carotenoids, or flavonoids—while fewer efforts are directed toward isolating, purifying, and characterizing individual compounds. This also includes a lack of studies investigating their specific biological effects, molecular mechanisms, or potential synergistic and antagonistic interactions. Furthermore, bibliometric analysis reveals that approximately 40% of publications for papaya and 60% for dragon fruit center around antioxidant activity, leaving a promising research avenue open for broader biological evaluations and potential physiological and clinical implications.

From a technical and innovation-oriented perspective, the application of modern and sustainable extraction techniques for recovering bioactive compounds from fruit by-products is gaining relevance. However, scientific research in this field remains limited, despite its alignment with global and European sustainability priorities. These include the European Union’s Green Deal (2019) and strategies like the “Sustainable Chemicals Strategy of the Union: Time to Deliver” (2021), which emphasize the importance of sustainable innovation, waste reduction, and circular economy approaches. In this context, the valorization of fruit by-products contributes to SDG 12 (Responsible Consumption and Production), fostering a more efficient and environmentally conscious agri-food system.

The bibliometric analysis conducted offers valuable insights into current research trends. For papaya, well-developed keyword networks point to extensive investigations of chemical composition but reveal a lack of in-depth biological assays. In contrast, pitahaya research shows a strong focus on antioxidant properties, particularly in the peel, while other bioactivities and associated compounds remain underexplored. Notably, the simple keyword structures associated with pitahaya seeds suggest minimal scientific attention, highlighting a highly promising area for future investigation, especially regarding their nutritional, functional, and pharmacological potential.

Moreover, the keyword co-occurrence maps generated through VosViewer offer more than just descriptive metrics—they enable the identification of thematic clusters and underexplored research avenues. This includes, for instance, the limited investigation of seed compounds, the scant use of circular economy approaches, or the insufficient application of advanced extraction methods. These visualizations provide a deeper interpretive layer that supports the prioritization of emerging topics and the design of future research agendas.

Overall, this review underscores the need for expanded interdisciplinary research efforts focused on underexplored by-products, such as pitahaya seeds, and on conducting comprehensive biological assays. Advancing knowledge in these areas will not only promote the sustainable use of tropical fruit residues but also enable the development of high-value functional ingredients, supporting both environmental goals and innovation within the circular economy. Furthermore, the valorization of food by-products not only contributes to sustainability and resource efficiency but also holds strong ethical value, as it promotes the responsible use of natural resources and reduces food waste.

## Figures and Tables

**Table 1:** The main research areas in papaya and dragon fruit.

Fruit	Research Area	Publications
Papaya	Plant Sciences	5574
	Agriculture	5235
	Food Science Technology	2451
	Biochemistry Molecular Biology	2820
	Environmental Sciences Ecology	2367
Dragon Fruit	Plant Sciences	43
	Agriculture	51
	Food Science Technology	31
	Physiology	25
	Genetics Heredity	24

**Table 2:** Principal journals and their metrics.

Fruit	Journal	Publications	IF	h	Q
Papaya	*Plant Disease*	121	4.8	128	Q1
	*Revista Brasileira de Fruticultura*	118	1.0	38	Q1
	*Hortscience*	107	1.8	94	Q2
	*Food Chemistry*	91	8.5	324	Q1
	*Phytopathology*	88	3.2	150	Q1
Dragon Fruit	*Scientia Horticulturae*	33	3.9	145	Q1
	*Plant Disease*	28	4.8	128	Q1
	*Food Chemistry*	23	8.5	324	Q1
	*Revista Brasileira de Fruitcultura*	21	1.0	38	Q4
	*Postharvest Biology and Technology*	20	6.4	170	Q1

(IF): impact factor of the journal; (h): h-index of the journal; (Q): quartile classification of the journal.

**Table 3:** Leading nations in papaya and dragon fruit research during the 2014–2024 phase.

Fruit	Country	Publications
Papaya	USA	1701
	India	1214
	Brazil	1073
	People’s Republic of China	624
	Mexico	473
Dragon fruit	People’s Republic of China	351
	Brazil	135
	Mexico	95
	Malaysia	90
	USA	61

## Data Availability

No new data were created or analyzed in this study.

## Abbreviations

The following abbreviations are used in this manuscript:

FAO	Food and Agriculture Organization
SDGs	Sustainable Development Goals
SCI-E	Science Citation Index Expanded
WOS	Web of Science
